# Association between glycemia risk index and carotid intima-media thickness in type 2 diabetes

**DOI:** 10.3389/fendo.2025.1563734

**Published:** 2025-06-19

**Authors:** Lingyun Zhao, Hongyan Heng, Qinyuan Xie, Chenghong Liang, Sijia Guo, Ziyi Zhang, Huijuan Yuan

**Affiliations:** ^1^ Department of Endocrinology, Zhengzhou University People's Hospital, Zhengzhou, China; ^2^ Department of Endocrinology, Henan Provincial Key Medicine Laboratory of Intestinal Microecology and Diabetes, Diabetes Microecology Diagnosis and Treatment and Transformation Engineering Research Center of Henan Province, Henan Provincial People’s Hospital, Zhengzhou, China

**Keywords:** glycemia risk index, type 2 diabetes, carotid intima-media thickness, cardiovascular diseases, continuous glucose monitoring

## Abstract

**Objective:**

To investigate the association between the Glycemic Risk Index (GRI) and carotid intima-media thickness (CIMT) in type 2 diabetes mellitus (T2DM) patients and evaluate the clinical utility of GRI for early vascular risk assessment.

**Methods:**

This retrospective study included 450 previously untreated patients with T2DM prior to hospitalization. We calculated GRI using CGM data and assessed CIMT with high-resolution ultrasound. Multiple linear and logistic regression analyses assessed the association between GRI and CIMT. Receiver operating characteristic (ROC) curve analyses evaluated GRI’s predictive performance.

**Results:**

There was a significant positive correlation between GRI and CIMT (r = 0.42, P < 0.001). After adjusting for confounders, GRI remained an independent predictor of CIMT thickening (OR = 7.226, 95% CI: 5.597–8.856, P < 0.001). ROC analysis revealed that GRI alone predicted abnormal CIMT with an AUC of 0.869.

**Conclusion:**

GRI is a robust marker for predicting CIMT thickening in T2DM patients, providing a novel approach for cardiovascular risk stratification. This study underscores the potential of integrating GRI into routine diabetes management to improve vascular outcomes.

## Introduction

Type 2 diabetes mellitus (T2DM) is a significant global public health challenge, with its prevalence projected to reach 12.2% by 2045, affecting approximately 783.2 million individuals worldwide (1). Cardiovascular disease (CVD) remains the leading cause of morbidity and mortality in T2DM, imposing a substantial economic burden (2). Persistent hyperglycemia-induced vascular endothelial damage is a core pathological mechanism driving diabetic vascular complications, accelerating atherosclerosis, arterial stiffness, and macrovascular dysfunction (3, 4).

Carotid intima-media thickness (CIMT) is a well-established, non-invasive marker of subclinical atherosclerosis and an independent predictor of cardiovascular events (5). Numerous longitudinal studies have validated CIMT as a robust and independent predictor of future cardiovascular events—particularly stroke and myocardial infarction—even after adjustment for conventional risk factors (6–8). In individuals with T2DM, elevated CIMT indicates subclinical atherosclerosis and is also associated with the advancement of microvascular and macrovascular complications. These findings underscore the prognostic value of CIMT and support its clinical utility as a surrogate marker for early vascular risk stratification and timely intervention in diabetic populations (9).

While optimal glycemic control is essential for mitigating vascular risk, traditional glycemic indices offer only a limited perspective on glucose dynamics. Glycated hemoglobin (HbA1c), although long considered the gold standard, reflects average glucose levels over several months and fails to capture short-term fluctuations or glycemic excursions that may trigger oxidative stress and vascular injury (10). Time-in-range (TIR), derived from continuous glucose monitoring (CGM), has gained popularity as a more dynamic indicator; however, it primarily reflects the proportion of time spent within a target glucose range and does not comprehensively quantify the burden of hypoglycemia or hyperglycemia (11).

The Glycemia Risk Index (GRI) is a novel composite metric that addresses these limitations by integrating the frequency, magnitude, and duration of hypo- and hyperglycemic episodes based on CGM data (12). Unlike HbA1c and TIR, GRI provides a continuous, risk-weighted measure of glycemic instability, capturing the full spectrum of glycemic excursions. Studies have demonstrated that GRI captures glucose variability and the associated metabolic burden more effectively than conventional indices. It has shown enhanced prognostic utility for identifying microvascular complications, such as retinopathy and albuminuria (13–15). More recently, a study by Cai et al. reported an independent association between elevated GRI and arterial stiffness, suggesting its potential relevance for macrovascular outcomes in T2DM (16). Furthermore, comparative analyses have shown that GRI outperforms traditional CGM-derived indices—such as TIR and the coefficient of variation—in identifying deleterious glycemic profiles associated with endothelial dysfunction and oxidative stress (17–19).

Despite this growing body of evidence supporting GRI as a valuable glycemic risk metric, its relationship with carotid structural alterations—particularly CIMT thickening—remains poorly characterized. Given that CIMT reflects early-stage atherosclerosis and is a robust predictor of stroke and myocardial infarction, elucidating its association with GRI may offer novel insights into the vascular consequences of glycemic instability and further refine cardiovascular risk assessment in diabetes care.

Therefore, this study aims to investigate the association between the Glycemia Risk Index (GRI) and carotid intima-media thickness (CIMT) in patients with type 2 diabetes mellitus (T2DM) to improve cardiovascular risk stratification and advance the clinical application of CGM-derived metrics in diabetes management.

## Methods

### Data source and study population

This study included 450 type 2 diabetes mellitus (T2DM) patients hospitalized in the Department of Endocrinology, Henan Provincial People’s Hospital, Zhengzhou University, from January 2019 to September 2021. Inclusion criteria were: (1) diagnosis of T2DM based on 1999 WHO criteria; (2) age ≥ge years; (3) no hypoglycemic drug use within 3 months before admission; (4) no history of severe cardiovascular disease, which was defined as prior myocardial infarction, or previous coronary revascularization procedures including percutaneous coronary intervention (PCI) or coronary artery bypass grafting (CABG); and (5) completion of continuous glucose monitoring (CGM) and carotid ultrasound. Exclusion criteria included: (1) type 1, special types, gestational, or unclear diabetes; (2) severe organ dysfunction, malignancy, or infection; and (3) incomplete CGM data (<3 days). The study was approved by the Ethics Committee of Henan Provincial People’s Hospital (NO. 2018048) and adhered to the 2008 Declaration of Helsinki.

### Clinical and biochemical information

Demographic and clinical data, including gender, age, BMI, diabetes duration, blood pressure, and smoking status, were collected. Biochemical analyses of fasting blood samples measured total cholesterol (TC), triglycerides (TG), LDL-C, HDL-C, and HbA1c. Urine samples were analyzed for creatinine to estimate the glomerular filtration rate (eGFR).

### Continuous glucose monitoring and glycemia risk index

CGM data were collected using the FreeStyle Libre-CGM system (Abbott). Sensors placed on the upper arm recorded blood glucose every 5 minutes for two weeks. Glycemic variability metrics included coefficient of variation (CV), mean amplitude of glycemic excursions (MAGE), largest amplitude of glycemic excursions (LAGE), and mean daily difference (MODD). Time below range (TBR), time above range (TAR), very-low glucose (VLow), low glucose (Low), high glucose (High), and very-high glucose (VHigh) were also recorded.

The GRI was calculated as follows (20–22):


$$\begin{array}{l} \text{Hypoglycemia}\;\text{component}\;=\;\text{VLow}\;+\;\left(0.8\;\times \;\text{Low}\right)\\ \text{Hyperglycemia}\;\text{component}\;=\;\text{VHigh}\;+\;\left(0.5\;\times \;\text{High}\right)\\ \text{GRI}\;=\;\left(3.0\;\times \;\text{hypoglycemia}\;\text{component}\right)\;+\;\left(1.6\;\times \;\text{hyperglycemia}\;\text{component}\right) \end{array}$$

### Carotid Doppler ultrasonography

Carotid Doppler ultrasonography conducted using an ACUSON Sequoia 512 device (10 MHz probe), measured CIMT 10 mm below the bifurcation of both carotid arteries. The average CIMT was calculated from both sides, with abnormal CIMT defined as ≥1.0 mm (6, 7).

### Statistical analysis methods

We divided participants into two groups based on CIMT (≥1.0 mm or <1.0 mm) and further stratified them by GRI quartiles. Continuous variables were tested for normality using the Kolmogorov-Smirnov test and reported as mean ± standard deviation or median (interquartile range). Categorical variables were summarized as frequency (percentage) and compared using t-tests, Mann-Whitney U tests, or χ2 tests, as appropriate.

Spearman correlation analysis assessed relationships between GRI, glycemic variability metrics, and CIMT. Linear regression explored the association between GRI and CIMT, adjusting for confounders across three models: Model 1 (unadjusted), Model 2 (adjusted for age and sex), and Model 3 (further adjusted for BMI, diabetes duration, smoking, SBP, TG, HDL-C, and LDL-C). Variance inflation factor (VIF) analysis confirmed no multicollinearity.

Binary logistic regression analyzed associations between GRI quartiles and CIMT thickening (≥ 1.0 mm), presenting odds ratios (OR) with 95% CI using the same three models. Subgroup analyses examined GRI-CIMT interactions by stratifying participants by sex, age (< 40, 40–50, ≥50 years), BMI (< 25, ≥25 kg/m²), and diabetes duration (≤3, >3 years), adjusting for confounders except for the stratification variable.

Receiver operating characteristic (ROC) curve analysis was used to evaluate the predictive performance of various glycemic metrics in identifying carotid intima-media thickening (CIMT ≥ 1.0 mm) (21). The area under the curve (AUC) was calculated for the glycemic risk index (GRI), HbA1c ≥ 7%, and time in range (TIR < 70%). GRI was analyzed as a continuous variable, and the optimal cutoff value was determined by maximizing Youden’s index. At this threshold, sensitivity, specificity, positive predictive value (PPV), and negative predictive value (NPV) were calculated. HbA1c and TIR were treated as binary variables based on clinical guideline recommendations.

We conducted all statistical analyses using R (v4.3.1), SPSS (v26), and GraphPad Prism (9.0), considering *P* < 0.05 statistically significant.

## Results

### Clinical features

The study included 450 participants, divided into two groups based on carotid intima-media thickness (CIMT): a normal CIMT group (n = 193) and an abnormal CIMT group (n = 257). **Table 1** summarizes the general clinical characteristics of the two groups. The abnormal CIMT group showed significantly higher GRI and glycemic variability metrics (CV, MAGE, MODD, LAGE). Additionally, the abnormal CIMT group demonstrated older age, higher BMI, greater hospitalization costs, and a higher prevalence of hypertension. No significant differences were observed between the groups for lipid levels (total cholesterol, triglycerides, HDL-C, LDL-C) or blood pressure. These findings suggest that GRI and glycemic fluctuations may play critical roles in CIMT thickening.

**Table 1:** Characteristics of study participants are analyzed according to CIMT status.

Characteristic	Total (n=450)	Normal CIMT(n=193)	Abnormal CIMT(n=257)	P-Value
Age, years	47(39, 54)	42 (34-51)	50 (45-56)	<0.001*
Duration of disease, years	3(1-5)	3(1-5)	3(1-5)	0.641
	13437.00 (10544.25 - 17800.50)			
BMI, kg/m^2^	25.25 (23.72 - 27.04)	25.25 (23.14,26.58)	25.25 (24.34,27.43)	0.003^*^
Systolic blood pressure, mmHg	130.00 (120.00 - 141.75)	130.00 (119.00-141.00)	130.00 (120.00-142.00)	0.334
Diastolic blood pressure, mmHg	80.00 (73.25 - 87.00)	80.00 (72.00-87.00)	80.00 (74.00-86.00)	0.722
Pulse rate	80.00 (75.00 - 85.00)	80.00 (74.00-85.00)	80.00 (75.00-84.00)	0.834
Total cholesterol, mmol/L	4.48 (3.79 - 5.20)	4.50 (3.78-5.29)	4.48 (3.83-5.14)	0.197
Triglycerides, mmol/L	1.69 (1.22 - 2.64)	1.69 (1.36-2.75)	1.69 (1.16-2.52)	0.034^*^
HDL cholesterol, mmol/L	1.05 (0.94 - 1.21)	1.05 (0.94-1.21)	1.05 (0.94-1.20)	0.94
LDL cholesterol, mmol/L	2.42 (1.92 - 3.02)	2.42 (1.90-3.15)	2.42 (1.93-2.93)	0.197
FPG, mmol/L	7.58 (5.99 - 9.48)	7.58 (5.96-9.17)	7.58 (6.00-9.66)	0.483
HbA1c (%)	8.10 (7.40 - 9.20)	8.10 (7.00-8.70)	8.10 (7.80-9.40)	0.001^*^
CV, %	31 (27-35)	28 (25-31)	33 (29-37)	<0.001*
MAGE, mmol/L	5.25 (4.38 - 6.32)	4.71 (3.87-5.54)	5.84 (4.81-6.85)	<0.001*
MODD, mmol/L	0.63 (0.48 - 0.78)	0.73 (0.61-0.89)	0.56 (0.42-0.71)	<0.001*
LAGE, mmol/L	13.05 (10.60 - 15.80)	11.70 (9.40-13.80)	14.40 (12.00-16.60)	<0.001*
TIR (3.9–10 mmol/L), %	81 (70 - 89)	90 (82-93)	74 (63-82 )	<0.001*
TBR (<3.9 mmol/L), %	1 (0 - 4)	1 (0-3)	2 (0-6)	<0.001*
TAR (>10 mmol/L), %	15 (6 - 27)	8 (4-16)	22 (11-33)	<0.001*
GRI, %		12 (8-20)	32 (22-45)	<0.001*
Scr, umol/L	58.00 (51.00-68.00)	58.00 (51.00-69.00)	58.00 (51.00-66.00)	0.554
Uric acid, umol/L	312.00 (256.00 - 364.75)	312.00 (269.00-372.00)	312.00 (247.00-354.00)	0.096
Urea to creatinine ratio	0.09 (0.09 - 0.11)	0.09 (0.08-0.10)	0.09 (0.09-0.11)	0.06
EGFR, mL/min	14.00 (14.00 - 14.00)	14.00 (14.00-14.00)	14.00 (14.00-14.00)	0.899
Male, n(%)				
Female	148 (32.89%)	126 (65.28%)	176 (68.48%)	0.48
Male	302 (67.11%)	67 (34.72%)	81 (31.52%)	
Current smoking, n (%)				
No	288 (64.00%)	74 (38.34%)	88 (34.24%)	0.374
YES	162 (36.00%)	119 (61.66%)	169 (65.76%)	
Alcohol consumption, n (%)				
No	293 (65.11%)	63 (32.64%)	94 (36.58%)	0.424
YES	157 (34.89%)	130 (67.36%)	163 (63.42%)	
Family history of genetic diseases, n (%)				
No	289 (64.22%)	68 (35.23%)	93 (36.19%)	0.843
YES	161 (35.78%)	125 (64.77%)	164 (63.81%)	
Hypertension ,n (%)				
No	235 (52.22%)	90 (46.63%)	125 (48.64%)	0.703
YES	215 (47.78%)	103 (53.37%)	132 (51.36%)	
Hyperlipidemia history, n (%)				
No	358 (79.56%)	49 (25.39%)	43 (16.73%)	0.025^*^
YES	92 (20.44%)	144 (74.61%)	214 (83.27%)	

Data for continuous variables were expressed as median (quartile distance), data for categorical variables as numerical (percentage) CIMT, carotid intima-media thickening; BMI, body mass index; HDL, high-density lipoprotein; LDL, low-density lipoprotein; FPG, fasting blood glucose; HbA1c, glycosylated hemoglobin; CV, coefficient of variation; MAGE, the average amplitude of blood sugar fluctuations; MODD: average daily difference; TIR, range of time; TAR, out-of-range time; GRI, glycemia risk index; TBR, time below range; LAGE, the maximum value of blood sugar fluctuations.

Then, to explore the relationship between different GRI levels and CIMT, participants were further divided into four groups (Q1–Q4) based on GRI quartiles (**Table 2**). Higher GRI levels were associated with significantly increased age, hospitalization costs, fasting plasma glucose (FPG), and HbA1c levels, along with elevated glycemic variability indices (CV, MAGE, MODD). However, lipid levels and other metabolic markers did not differ significantly across quartiles. Similarly, clinical features such as smoking history, alcohol consumption, family history of genetic disease, hypertension, and hyperlipidemia were not significantly different. Further analyses indicated that the prevalence of carotid intimal thickening increased progressively with GRI quartiles (*P* < 0.001, **Supplementary Figure S1**).

**Table 2:** Study participants were grouped according to GRI quartile characteristics.

Characteristic	GRI,%				P
	Q1(n=113) (0.0081,0.7947)	Q2(n=112) (0.81, 12.58)	Q3(n=112) (12.58, 22.52)	Q4(n=113) (22.52, 34.84)	
Age, years	46.26 ± 10.36	45.68 ± 11.19	45.44 ± 10.75	50.01 ± 9.41	0.003^*^
Duration of disease, years	3(1-5)	3(1-5)	3(1-5)	3(1-5)	0.914
Cost, RMB	12616.00 (8747.00 - 16564.00)	13437.00 (10929.75 - 16726.00)	12795.50 (10370.75 -17499.75)	15711.00 (12420.00 -20107.00)	<0.001*
BMI, kg/m^2^	25.25 (25.56, 27.25)	25.25 (24.43, 27.70)	25.25 (24.13, 26.30)	25.25 (23.08, 26.42)	0.355
Systolic blood pressure, mmHg	130.00 (120.00 - 141.00)	129.00 (120.00 - 140.25)	130.00 (120.00 - 140.00)	131.00 (122.00 - 146.00)	<0.001*
Diastolic blood pressure, mmHg	80.00 (73.00 - 90.00)	80.00 (74.00 - 88.00)	79.00 (72.00 - 85.00)	80.00 (74.00 - 86.00)	<0.001*
Pulse rate	80.00 (76.00 - 85.00)	80.00 (74.00 - 84.25)	80.00 (75.00 - 84.00)	80.00 (75.00 - 86.00)	<0.001*
Total cholesterol, mmol/L	4.48 (3.69 - 5.29)	4.62 (3.93 - 5.21)	4.48 (3.77 - 5.13)	4.48 (3.91 - 5.10)	<0.001*
Triglycerides, mmol/L	1.72 (1.36 - 2.86)	1.73 (1.42 - 2.73)	1.58 (1.16 - 2.42)	1.69 (1.06 - 2.07)	<0.001*
HDL cholesterol, mmol/L	1.05 (0.92 - 1.22)	1.05 (0.96 - 1.16)	1.04 (0.92 - 1.21)	1.06 (0.95 - 1.26)	<0.001*
LDL cholesterol, mmol/L	2.42 (1.80 - 3.18)	2.42 (1.98 - 3.08)	2.42 (1.93 - 2.94)	2.42 (1.91 - 2.97)	<0.001*
CIMT, mm	0.90 (0.80 - 0.90)	0.90 (0.80 - 1.10)	1.10 (0.90 - 1.10)	1.20 (1.10 - 1.20)	<0.001*
FPG, mmol/L	7.47 (5.70 - 8.20)	7.58 (6.37 - 10.24)	7.58 (6.04 - 8.99)	7.58 (6.24 - 10.50)	<0.001*
HbA1c, %	7.90 (6.50 - 8.10)	8.10 (7.47 - 9.20)	8.10 (7.80 - 9.12)	8.10 (8.10 - 9.90)	<0.001*
CV, %	26 (22 - 29)	30 (27 - 33)	33 (30 - 36)	36 (31 - 40)	<0.001*
MAGE, mmol/L	4.13 (3.54 - 4.82)	5.15 (4.59 - 5.95)	5.83 (4.80 - 6.55)	6.43 (5.49 - 7.78)	<0.001*
MODD, mmol/L	0.82 (0.72 - 0.96)	0.65 (0.55 - 0.74)	0.59 (0.47 - 0.71)	0.43 (0.35 - 0.59)	<0.001*
LAGE, mmol/L	10.00 (8.70 - 12.20)	13.10 (11.03 - 14.62)	13.85 (12.40 - 15.80)	16.20 (13.10 - 18.50)	<0.001*
TIR (3.9–10 mmol/L), %	93 (90 - 96)	84 (80 - 88)	75 (71 - 82)	61 (52 - 67)	<0.001*
TBR (<3.9 mmol/L), %	1 (0 - 2)	1 (0 - 3)	2 (0- 6)	3 (1 - 9)	<0.001*
TAR (>10 mmol/L), %	6 (3 - 9)	15 (9 - 20)	23 (11 - 28)	36 (22 - 45)	<0.001*
Scr, umol/L	58.00 (51.00 - 66.00)	58.00 (50.75 - 68.00)	57.00 (48.00 - 65.00)	59.00 (52.00 - 72.00)	<0.001*
Uric acid, umol/L	312.00 (281.00 - 376.00)	312.00 (260.50 - 358.75)	312.00 (243.75 - 350.25)	311.00 (242.00 - 369.00)	<0.001*
Urea to creatinine ratio	0.09 (0.08 - 0.10)	0.09 (0.09 - 0.11)	0.09 (0.09 - 0.11)	0.09 (0.09 - 0.10)	<0.001*
EGFR, mL/min	14.00 (14.00 - 14.00)	14.00 (14.00 - 14.00)	14.00 (14.00 - 14.00)	14.00 (14.00 - 14.00)	<0.001*
Sex, n(%)					
Female	37 (32.74%)	36 (32.14%)	40 (35.71%)	35 (30.97%)	0.891
Male	76 (67.26%)	76 (67.86%)	72 (64.29%)	78 (69.03%)	0.891
Current smoking, n(%)					
No	72 (63.72%)	68 (60.71%)	75 (66.96%)	73 (64.60%)	0.808
YES	41 (36.28%)	44 (39.29%)	37 (33.04%)	40 (35.40%)	0.808
Alcohol consumption, n (%)					
No	66 (58.41%)	77 (68.75%)	76 (67.86%)	74 (65.49%)	0.352
YES	47 (41.59%)	35 (31.25%)	36 (32.14%)	39 (34.51%)	0.352
Family history of genetic diseases, n (%)					
No	72 (63.72%)	73 (65.18%)	72 (64.29%)	72 (63.72%)	0.995
YES	41 (36.28%)	39 (34.82%)	40 (35.71%)	41 (36.28%)	0.995
Hypertension, n (%)					
No	64 (56.64%)	57 (50.89%)	59 (52.68%)	55 (48.67%)	0.673
YES	49 (43.36%)	55 (49.11%)	53 (47.32%)	58 (51.33%)	0.673
Hyperlipidemia history, n (%)					
No	87 (76.99%)	89 (79.46%)	89 (79.46%)	93 (82.30%)	0.806
YES	26 (23.01%)	23 (20.54%)	23 (20.54%)	20 (17.70%)	0.806
	(0.81, 12.58)	(12.58, 22.52)	(22.52, 34.84)	(34.84, 79.47)	

Data for continuous variables are expressed as median (interquartile distance), age is expressed as mean SD categorical variables are expressed as numerical (percentage) CIMT, carotid intima-media thickening; BMI, body mass index; HDL, high-density lipoprotein; LDL, low-density lipoprotein; FPG, fasting blood glucose; HbA1c, glycosylated hemoglobin; CV, coefficient of variation; MAGE, the average amplitude of blood sugar fluctuations; MODD: average daily difference; TIR, range of time; TAR, out-of-range time; GRI, blood glucose risk index; TBR, time below range; LAGE, the maximum value of blood sugar fluctuations.

### Correlation between CIMT and GRI

Spearman correlation analysis (**Supplementary Table S2**) found a significant positive correlation between CIMT and GRI, in addition, CIMT was also correlated with other indicators of blood glucose fluctuation. Multiple linear regression analysis (**Table 3**) confirmed GRI as an independent predictor of CIMT across all models. In the fully adjusted model (Model 3), GRI showed a strong association with CIMT (β = 0.488, 95% CI: 0.413–0.563, *P* < 0.001). Age was also positively associated with CIMT (β = 0.022, 95% CI: 0.018–0.026, *P* < 0.001), while gender, BMI, diabetes duration, lipid indices, and smoking history were not significantly associated (*P* > 0.05). These findings underline the importance of GRI as a robust marker of CIMT thickening.

**Table 3:** Multivariate linear regression analysis of the relationship between GRI and CIMT.

Variable	Model 1		Model 2		Model 3	
	β (95% CI)	P-value	β (95% CI)	P-value	β (95% CI)	P-value
const	-0.006 (-0.149, 0.137)	0.936	-0.006 (-0.149, 0.137)	0.936	-0.006 (-0.149, 0.137)	0.936
GRI	0.488 (0.413, 0.563)	<0.001*	0.488 (0.413, 0.563)	<0.001*	0.488 (0.413, 0.563)	<0.001*
Age			0.006 (0.005, 0.007)	<0.001*	0.006 (0.005, 0.007)	<0.001*
Sex			0.025 (-0.008, 0.059)	0.135	0.025 (-0.008, 0.059)	0.135
BMI					0.022 (0.018, 0.026)	<0.001*

The data were expressed as regression coefficient (95% CI) Model 1 adjusted for sex and age Model 2 further adjusted for BMI diabetes course current smoking status triglyceride HDL cholesterol and LDL cholesterol CIMT, carotid indium-media thickened; GRI, blood glucose risk index; HDL, high-density lipoprotein; LDL, low-density lipoprotein; BMI, body mass index.

### Logistic regression analysis

Logistic regression analysis further evaluated the relationship between GRI quartiles and CIMT thickening risk (**Table 4**). After adjusting for confounders (Model 3), participants in the higher GRI quartiles had significantly increased odds of CIMT thickening compared to the lowest quartile (Q2: OR = 2.631, 95% CI: 1.734–3.528, P < 0.001; Q3: OR = 4.675, 95% CI: 3.636–5.714, P < 0.001; Q4: OR = 7.226, 95% CI: 5.597–8.856, P < 0.001). Age (β = 0.136, 95% CI: 0.097–0.174, P < 0.001) and BMI (β = 0.347, 95% CI: 0.218–0.477, P < 0.001) were also significantly associated with CIMT thickening, whereas gender showed no significant association. These results highlight the relationship between GRI and CIMT risk, suggesting the potential utility of GRI in identifying at-risk populations.

**Table 4:** logistic regression analysis of different GRI levels and CIMT thickening.

Variable	Model 1		Model 2		Model 3	
	β (95% CI)	P-value	β (95% CI)	P-value	β (95% CI)	P-value
const	-2.040 (-2.618, -1.462)	<0.001*	-8.890 (-10.907, -6.873)	<0.001*	-18.451 (-23.205, -13.698)	<0.001*
Q1		<0.001*	<0.001*		<0.001*	
Q2	1.897 (1.210, 2.584)	<0.001*	2.483 (1.677, 3.288)	<0.001*	2.631 (1.734, 3.528)	<0.001*
Q3	3.092 (2.376, 3.808)	<0.001*	4.175 (3.257, 5.094)	<0.001*	4.675 (3.636, 5.714)	<0.001*
Q4	5.345 (4.192, 6.498)	<0.001*	6.090 (4.790, 7.390)	<0.001*	7.226 (5.597, 8.856)	<0.001*
Age			0.130 (0.096, 0.165)	<0.001*	0.136 (0.097, 0.174)	<0.001*
Sex			0.343 (-0.254, 0.939)	0.260	0.279 (-0.518, 1.077)	0.493
BMI					0.347 (0.218, 0.477)	<0.001*

The data were expressed as regression coefficient (95% CI) Model 1 adjusted for sex and age Model 2 further adjusted for BMI diabetes course current smoking status triglyceride HDL cholesterol and LDL cholesterol CIMT, carotid indium-media thickened; GRI, blood glucose risk index; HDL, high-density lipoprotein; LDL, low-density lipoprotein; BMI, body mass index.

### Subgroup analysis

**Figure 1:**
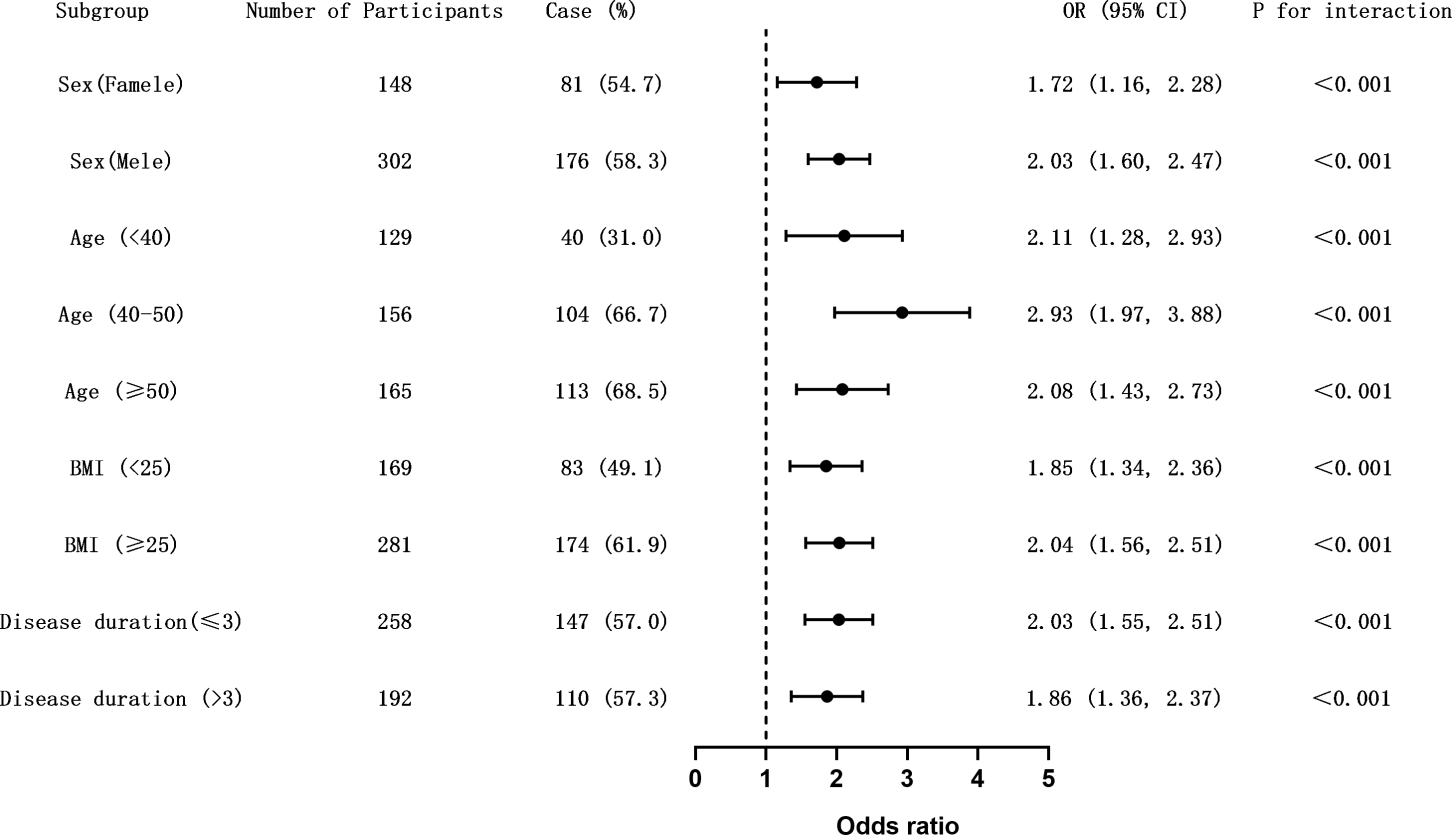
Subgroup analysis of the association between GRI and carotid intima-media thickness (CIMT) thickening. Forest plot showing odds ratios (ORs) and 95% confidence intervals (CIs) for the association between elevated glycemia risk index (GRI) and CIMT thickening (CIMT ≥ 1.0 mm) across predefined clinical subgroups. Horizontal lines represent 95% CIs. The dashed vertical line indicates the null value (OR = 1.0). Interaction p-values were calculated to assess effect modification. The association remained consistent across all subgroups without evidence of effect heterogeneity. **Figure 1**. Subgroup analysis of the association between GRI and carotid intima-media thickness (CIMT) thickening. Forest plot showing odds ratios (ORs) and 95% confidence intervals (CIs) for the association between elevated glycemia risk index (GRI) and CIMT thickening (CIMT ≥ 1.0 mm) across predefined clinical subgroups. Horizontal lines represent 95% CIs. The dashed vertical line indicates the null value (OR = 1.0). Interaction p-values were calculated to assess effect modification. The association remained consistent across all subgroups without evidence of effect heterogeneity.

Since age, BMI, and disease duration are recognized as risk factors for CIMT thickening, these variables were included in the regression model as interaction terms for analysis. The results (**Supplementary Table S3**) indicated no significant interactions between GRI and variables such as gender, age, BMI, and disease duration in predicting CIMT thickening. However, further subgroup analysis (**Figure 1**) revealed significant differences in the association between GRI and CIMT thickening across various demographic and clinical subgroups.

Male patients exhibited a higher odds ratio (OR = 2.03, 95% CI: 1.60–2.47) compared to females (OR = 1.72, 95% CI: 1.16–2.28), suggesting a stronger association between GRI and CIMT thickening in men. Among age groups, the 40–50 age group showed the highest risk (OR = 2.93, 95% CI: 1.97–3.88), followed by patients aged ≥50 years (OR = 2.08, 95% CI: 1.43–2.73). For BMI, patients with BMI ≥25 kg/m² had a significantly greater risk of CIMT thickening (OR = 2.04, 95% CI: 1.56–2.51) compared to those with BMI <25 kg/m² (OR = 1.85, 95% CI: 1.34–2.36).

The influence of diabetes duration was relatively smaller, with patients having a disease duration ≤3 years showing a slightly higher odds ratio (OR = 2.03, 95% CI: 1.55–2.51) compared to those with >3 years (OR = 1.86, 95% CI: 1.36–2.37). These findings underscore the importance of gender, age, and BMI as critical stratified factors influencing the risk of CIMT abnormalities. Meanwhile, the association between GRI and CIMT thickening remains significant across different diabetes duration groups, highlighting its robust predictive value.

### ROC curve analysis

**Figure 2:**
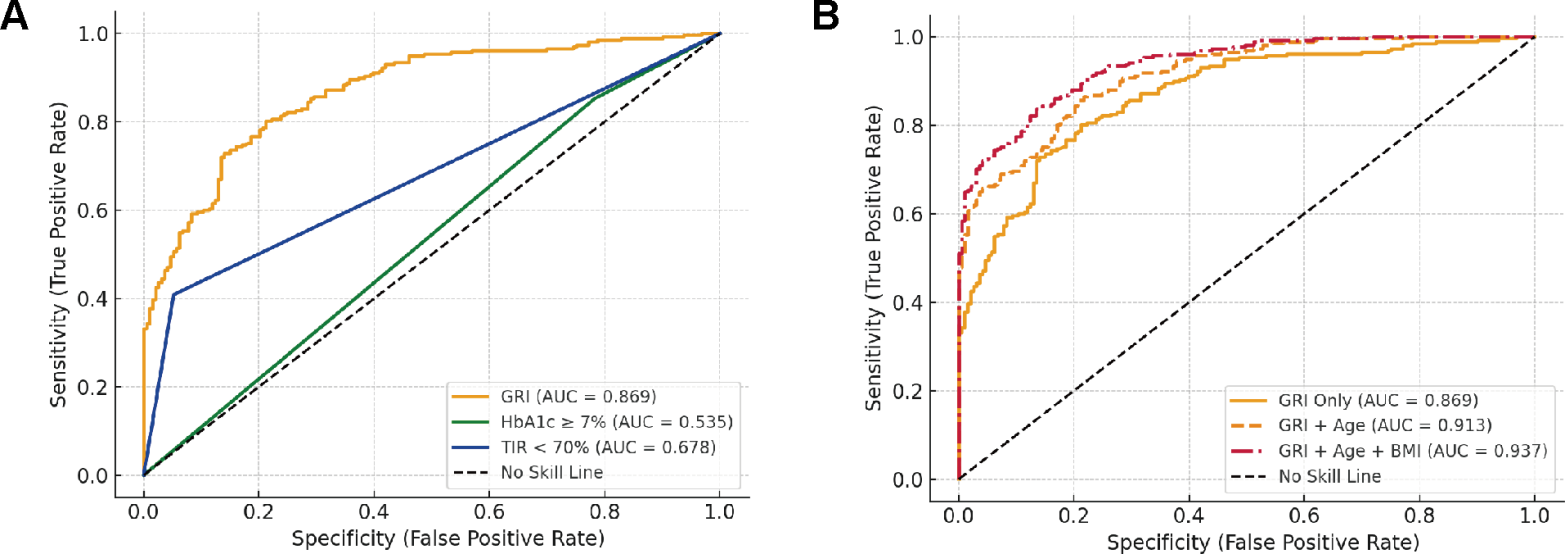
Receiver operating characteristic (ROC) curves for predicting CIMT thickening. **(A)** Comparative diagnostic performance of glycemic metrics including the Glycemia Risk Index (GRI), HbA1c ≥ 7%, and Time in Range (TIR < 70%) in identifying CIMT thickening (CIMT ≥ 1.0 mm). **(B)** Predictive performance of GRI alone and in combination with traditional risk factors.

To evaluate the diagnostic performance of GRI and other glycemic indices for predicting carotid intima-media thickness (CIMT ≥ 1.0 mm), receiver operating characteristic (ROC) curve analysis was conducted (**Figure 2A**; **Supplementary Table S4**). Among the individual markers, GRI demonstrated the highest discriminative ability, with an area under the curve (AUC) of 0.869, sensitivity of 0.802, and specificity of 0.788. In contrast, HbA1c ≥ 7% and TIR < 70% showed inferior performance, with AUCs of 0.495 and 0.678, respectively. While HbA1c exhibited high sensitivity (0.872), its specificity was markedly low (0.119); conversely, TIR had high specificity (0.948) but poor sensitivity (0.409), indicating imbalanced classification performance.

To further explore the incremental value of clinical variables, we constructed combined models incorporating traditional risk factors. As shown in **Figure 2B**, the combination of GRI and Age increased the AUC to 0.913, and the addition of BMI further improved the AUC to 0.937. Notably, the positive predictive value (PPV) also increased from 0.834 (GRI alone) to 0.892 (GRI + Age + BMI), indicating a substantial gain in classification robustness. These results suggest that integrating GRI with conventional clinical parameters significantly enhances its predictive value for subclinical atherosclerosis in patients with type 2 diabetes.

## Discussion

This study evaluated the clinical applicability of the Glycemic Risk Index (GRI) for predicting subclinical macrovascular complications in individuals with type 2 diabetes mellitus (T2DM). Although prior studies have demonstrated that GRI is significantly associated with microvascular complications such as diabetic retinopathy and proteinuria (12, 14), its role in macrovascular pathology remains insufficiently studied. Our results show that elevated GRI levels are significantly associated with increased carotid intima-media thickness (CIMT), suggesting its potential utility as a noninvasive, integrative biomarker for early cardiovascular risk assessment in patients with T2DM.

These findings align with a Japanese cohort study, which examined the association between GRI and various markers of atherosclerosis. While no significant relationship was observed between GRI and mean CIMT, the study identified strong associations between GRI and longer diabetes duration, higher HbA1c levels, elevated mean glucose concentrations, brachial-ankle pulse wave velocity (baPWV), and the gray-scale median (GSM) of the carotid artery wall—even after adjustment for conventional cardiovascular risk factors (22). The authors proposed that GRI may reflect vascular remodeling and overall atherosclerotic burden beyond arterial wall thickness alone. By focusing on CIMT as a structural indicator of subclinical atherosclerosis, our study extends the evidence base for GRI as a predictor of early macrovascular changes. This complementary relationship may reflect differences in population characteristics, disease stages, endpoint definitions, or methods of GRI calculation.In addition, another investigation found an inverse and independent association between Time in Range (TIR)—a CGM-derived glycemic variability metric—and CIMT, reinforcing the relevance of CGM-based parameters in vascular risk evaluation (23). Compared with single glycemic indices such as TIR and HbA1c, GRI integrates the burden of both hypo- and hyperglycemia, offering a more holistic measure of glycemic instability.

Previous studies have demonstrated that intermittent hyperglycemia activates the NADPH oxidase system, leading to excessive production of reactive oxygen species (ROS), which in turn reduces the bioavailability of nitric oxide (NO) and impairs endothelial vasodilation (19, 24). In parallel, ROS also triggers the NF-κB signaling cascade, resulting in the upregulation of pro-inflammatory cytokines (e.g., IL-6 and TNF-α), enhanced leukocyte adhesion, endothelial apoptosis, and foam cell formation—all hallmark processes in the development of atherosclerosis (25).As an integrated measure of overall glycemic variability, an elevated GRI may exacerbate metabolic disturbances and amplify oxidative and inflammatory responses, thereby accelerating macrovascular injury in patients with diabetes mellitus; (26).

Subgroup analyses suggested that sex, age, and body mass index (BMI) may significantly influence the relationship between GRI and CIMT. Men are more prone to insulin resistance and dyslipidemia due to the absence of estrogen-mediated vascular protection (27–29), while postmenopausal women may experience accelerated vascular aging owing to hormonal decline (30, 31);. Additionally, aging-related β-cell dysfunction and arterial stiffness further heighten the risk of atherosclerosis (32);. Obesity, a key component of metabolic syndrome, may promote CIMT progression via persistent low-grade inflammation and endothelial dysfunction (29, 33);.Because GRI dynamically captures fluctuations in both hyperglycemia and hypoglycemia, it may offer greater clinical value than traditional static glycemic measures such as HbA1c. Regular monitoring of GRI—at intervals of 3 to 6 months—could facilitate earlier cardiovascular risk stratification, particularly in high-risk individuals such as those with inadequate glycemic control, obesity, or advanced age. Persistent elevations in GRI may act as early warning signals, prompting clinicians to intensify glucose-lowering therapy, initiate statin treatment, or recommend lifestyle interventions in a timely, personalized manner.

This study has several limitations. First, as a single-center retrospective analysis, selection bias and missing data may affect the generalizability of our findings. Moreover, the GRI algorithm has not yet been standardized, and the absence of consensus thresholds for cardiovascular risk stratification may compromise inter-study comparability and reproducibility. Future multicenter, prospective, and interventional trials are essential to validate the predictive robustness of GRI and to better define its role in guiding cardiovascular risk management.Second, we did not assess lifestyle variables such as diet, physical activity, or sleep behaviors, which may confound the observed association between GRI and CIMT. Future research should integrate standardized lifestyle questionnaires or wearable technologies to better account for behavioral confounders, improve model accuracy, and support more rigorous causal inference.Third, this study did not stratify female participants based on menopausal status, potentially underestimating the effects of hormonal changes on vascular risk. Future studies should consider including sex hormone measurements and menopausal classification to improve the precision of sex-specific risk assessments.

## Conclusion

This study identifies GRI as an independent predictor of CIMT thickening in type 2 diabetes. As a noninvasive, CGM-derived metric, GRI may enable early identification of high-risk patients and support timely, individualized interventions—such as optimizing glycemic control or initiating statin therapy—to slow the progression of subclinical atherosclerosis.

## Data Availability

The datasets used and/or analysed during the current study available from the corresponding author on reasonable request. Requests to access these datasets should be directed to HY, hjyuan@zzu.edu.cn.
