# Supplementary material for: Association between glycemia risk index and carotid intima-media thickness in type 2 diabetes
Source: Front Endocrinol (Lausanne). 2025 Jun 19;16:1563734. doi: 10.3389/fendo.2025.1563734 (PMC12224650; doi:10.3389/fendo.2025.1563734)
Supplement: Supplementary file 1 [file DataSheet1.docx]

**Figure S1**


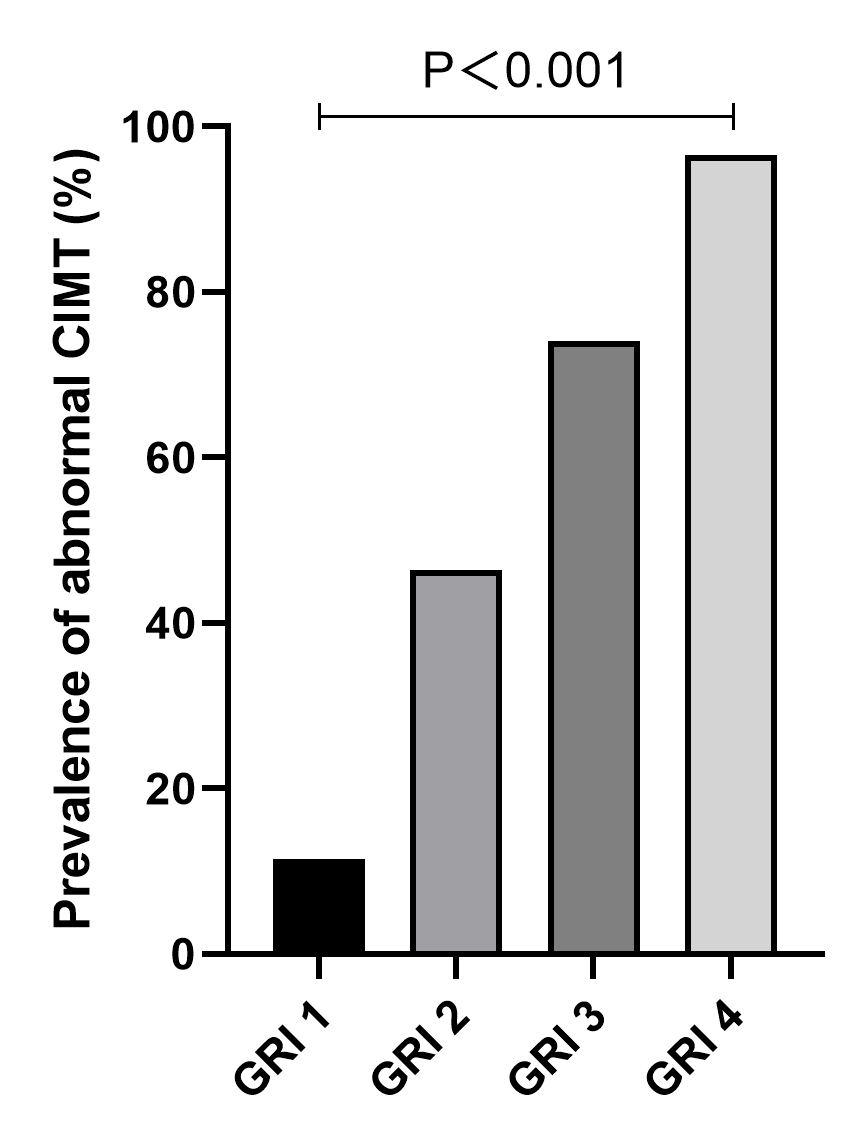


Prevalence of CIMT thickening in GRI quartile (Q1 Q4) GRI 1:0.81 12.58,GRI 2:12.58 22.52,GRI 3:22.52 34.84,GRI 4:34.84 79.47; CIMT, carotid intima-media thickening; GRI, glycemic risk index.

| Variable | VIF |
| --- | --- |
| GRI, % | 1.047834346 |
| Age, years | 1.061705189 |
| Sex | 1.453584747 |
| BMI, kg/m^2^ | 1.05829385 |
| Duration of disease, years | 1.035527363 |
| Total cholesterol, mmol/L | 2.487504985 |
| Triglycerides, mmol/L | 1.138989456 |
| HDL cholesterol, mmol/L | 1.111650824 |
| LDL cholesterol, mmol/L | 2.325861882 |
| smoking | 1.37273072 |

**Table S1 Results of Variance inflation factor**

GRI, blood glucose risk index; BMI, body mass index; HDL, high-density lipoprotein; LDL, low-density lipoprotein; HDL, high-density lipoprotein; LDL, low-density lipoprotein

**Table S2 Spearman correlation analysis of the relationship between GRI and CIMT**

| Feature | Spearman Correlation | P-value |
| --- | --- | --- |
| GRI, % | 0.627 | 0.000^*^ |
| TBR(<3.9 mmol/L), % | 0.210 | 0.000^*^ |
| TAR(>10 mmol/L), % | 0.427 | 0.000^*^ |
| TIR (3.9–10 mmol/L), % | -0.574 | 0.000^*^ |
| CV, % | 0.433 | 0.000^*^ |
| LAGE, mmol/L | 0.344 | 0.000^*^ |
| MODD, mmol/L | -0.387 | 0.000^*^ |
| MAGE, mmol/L | 0.366 | 0.000^*^ |
| HbA1c (%) | 0.166 | 0.000^*^ |
| FBG, mmol/L | 0.028 | 0.552 |

GRI, blood glucose risk index; TBR, time below range; TAR, out-of-range time; TIR, range of time; CV, coefficient of variation; LAGE, the maximum value of blood sugar fluctuations; MODD: average daily difference; MAGE, the average amplitude of blood sugar fluctuations; HbA1c, glycosylated hemoglobin;FPG, fasting blood glucose;

| Interaction Variable | Interaction Coefficient | OR (95% CI) | P-value |
| --- | --- | --- | --- |
| Sex | 0.289 | 1.335 (0.698, 2.553) | 0.382 |
| Age, years | 0.097 | 1.101 (0.701, 1.731) | 0.675 |
| BMI, kg/m^2^ | 0.047 | 1.048 (0.562, 1.953) | 0.883 |
| Disease Duration, years | -0.189 | 0.827 (0.449, 1.526) | 0.544 |

**Table S3 Interactive validation**

BMI, body mass index

**Table S4 Diagnostic efficacy table of each index**

| Marker | AUC | Optimal Cutoff | Sensitivity | Specificity | PPV | NPV |
| --- | --- | --- | --- | --- | --- | --- |
| GRI | 0.869 | 0.205 | 0.802 | 0.788 | 0.834 | 0.749 |
| HbA1c ≥ 7% | 0.495 | ≥ 7% | 0.872 | 0.119 | 0.569 | 0.411 |
| TIR < 70% | 0.678 | < 70% | 0.409 | 0.948 | 0.913 | 0.546 |
| GRI + Age | 0.913 | 0.492 | 0.856 | 0.793 | 0.846 | 0.805 |
| GRI + Age + BMI | 0.937 | 0.548 | 0.837 | 0.865 | 0.892 | 0.799 |

GRI, glycemia risk index; TIR, time in range; AUC, area under the receiver operating characteristic (ROC) curve; PPV, positive predictive value; NPV, negative predictive value.Note: The optimal cutoff for GRI was determined using Youden’s index.
